# Novel perspectives on stomatal impressions: Rapid and non-invasive surface characterization of plant leaves by scanning electron microscopy

**DOI:** 10.1371/journal.pone.0238589

**Published:** 2020-09-03

**Authors:** William J. Matthaeus, Jonathan Schmidt, Joseph D. White, Bernd Zechmann

**Affiliations:** 1 Department of Biology, Baylor University, Waco, Texas, United States of America; 2 Center for Microscopy and Imaging, Baylor University, Waco, Texas, United States of America; Chinese Academy of Sciences, CHINA

## Abstract

Scanning electron microscopy (SEM) is widely used to investigate the surface morphology, and physiological state of plant leaves. Conventionally used methods for sample preparation are invasive, irreversible, require skill and expensive equipment, and are time and labor consuming. This study demonstrates a method to obtain *in vivo* surface information of plant leaves by imaging replicas with SEM that is rapid and non-invasive. Dental putty was applied to the leaves for 5 minutes and then removed. Replicas were then imaged with SEM and compared to fresh leaves, and leaves that were processed conventionally by chemical fixation, dehydration and critical point drying. The surface structure of leaves was well preserved on the replicas. The outline of epidermal as well as guard cells could be clearly distinguished enabling determination of stomatal density. Comparison of the dimensions of guard cells revealed that replicas did not differ from fresh leaves, while conventional sample preparation induced strong shrinkage (-40% in length and -38% in width) of the cells when compared to guard cells on fresh leaves. Tilting the replicas enabled clear measurement of stomatal aperture dimensions. Summing up, the major advantages of this method are that it is inexpensive, non-toxic, simple to apply, can be performed in the field, and that results on stomatal density and *in vivo* stomatal dimensions in 3D can be obtained in a few minutes.

## Introduction

Scanning electron microscopy (SEM) is widely used to characterize the surface of plant leaves. It is a valuable tool to investigate the morphology of trichomes, stomatal density (SD), epicuticular waxes, accumulation of nano-particles, and infection with pathogens [1–9]. Biological samples for SEM have to be extensively prepared to withstand the vacuum and the high energy of the beam. Sample preparation is usually invasive, irreversible, requires skill and expensive equipment, and is time and labor consuming. Leaves have to be cut off, sectioned into small pieces, fixed, dehydrated, critical point dried, and sputter coated before they can be investigated by SEM at room temperature [2, 6, 8]. Sample preparation can take several days and results in artifacts such as shrinking of sensitive structures [2, 3, 5, 10, 11]. When SEM is applied in field studies the samples have to be prepared and transported to the lab, often under sub-optimal conditions that can lead to artifacts.

Alternatively, samples can be cryo-fixed, sputter coated, and imaged by SEM with a cryo-stage. Though this technique reduces the effects of shrinkage, such investigations require expensive equipment, high skill levels, cannot be performed in the field and induce other artifacts such as cracking of the surface [2, 3, 12]. Detached leaves can also be imaged, without further preparation, under low vacuum conditions or by environmental scanning electron microscopy (ESEM) closer to the atmospheric state [1, 5, 12, 13]. Nevertheless, the sample is still exposed to a low vacuum and high evaporation potential, which can induce artifacts on sensitive structures such as trichomes and stomata [2, 3, 12, 14]. In either high or low vacuum, leaves have to be detached from the plant and sectioned into small pieces if the whole leaf does not fit into the chamber of the SEM.

The above described conventionally used methods to study the surface characteristics of leaves using SEM, therefore, are arduous, expensive, and may not characterize the *in vivo* leaf state. They involve lengthy sample preparation procedures, expensive equipment, high levels of skill, and are time and labor consuming [2, 3, 12]. A powerful alternative to the methods described above is the preparation of replicas or impressions of leaves by using nail polish, rubber, glue, or silicone and the investigation with light microscopy [15–19] and SEM [20–23], which allows for *in vivo* physiological inference, for example of stomatal conductance. However, depending on the technique and material, the preparation of the replicas for SEM takes between 2 hours [23] and 1 week [20]. While these methods require less skill, time and labor they still require basic lab equipment such as light microscopes, ovens, and sputter coaters [17, 20–23] which makes them unsuitable for rapid investigations of the plant tissue and sample preparations in the field. Further, some replica materials damaged or killed the tissue [17, 19] or required the removal of leaves from the plants [20]. Additionally, some results were negatively affected by the use of organic solvents, material viscosity, environmental humidity, and mechanical damage [23]. For these reasons, leaf replica methods are not widely used for investigations of plant material in the SEM. This study demonstrates an easy to use, inexpensive method based on the preparation of leaf replicas that can be applied in the field to characterize the surface of leaves by SEM. We are the first to demonstrate that this method is non-invasive, allowing for the creation of time-series, or use on rare plants. Further, we introduce a novel perspective on how the 3-dimensionality of impressions and modern SEM can capture *in vivo* stomatal aperture characteristics.

## Materials and methods

### Plant growth and sampling conditions

**Fig 1:**
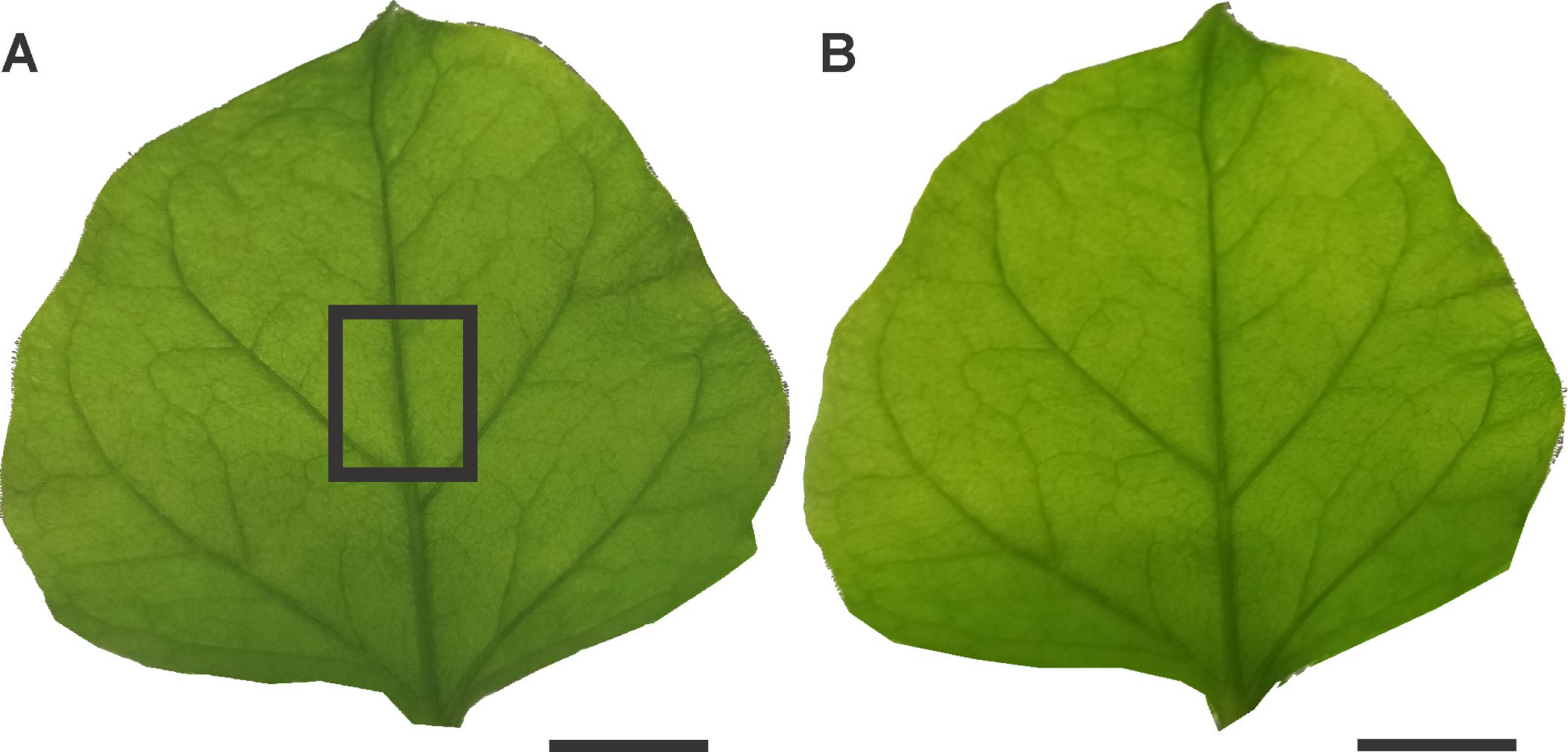
Photographs of plant leaves. Pictures show leaves (A) before and (B) immediately after the application of the putty. The photographs show youngest fully developed leaves at Day 1. The black box indicates the leaf area from which samples were taken for the investigations. Bars = 1 cm.

*Nicotiana benthamiana* were grown in a climate chamber with day/night temperatures of 22/26°, minimum/maximum relative humidity of 65/75%, soil moisture of 20/40%, a photosynthetic photon flux density of 55 μmol∙m^-2^∙s^-1^ and light/dark cycle 14/10 hours. Plants were grown in Sunshine® Mix #1 (SunGro Horticulture, Agawam, MA) with Osmocote Classic 14-14-14 slow release fertilizer. The youngest fully developed leaves were selected for this experiment and were investigated at Day 1 and 14 days later (Day 14) when they were fully developed adult leaves. Investigated leaves were the same size and the replicas were taken from the middle of the leaf close to the midrib as demonstrated by the black box in Fig 1A.

### Leaf replicas and photographs

Leaf replicas were made of the abaxial surface using Colténe PRESIDENT light body dental putty (Colténe/Whaledent AG, Alstätten, Switzerland) 30 minutes after the onset of light. Replicas were made by mixing 125 mg each, of base and catalyst in a plastic petri dish. Within 30 seconds of mixing, leaves were pressed onto the putty with even pressure for 5 minutes. The replicas were then pulled off from the leaf surface, mounted on aluminum stubs with carbon tape and imaged with a Hitachi TM3030Plus SEM (Hitachi High Technologies America, IL, USA) under low vacuum conditions and a FEI Versa 3D SEM (FEI, Hillsboro, OR, USA) under high vacuum conditions. For the latter replicas were sputter coated with 10 nm or iridium with a Leica EM ACE 600 sputter coater (Leica Microsystems, Wetzlar, Germany). SD was measured on replicas using ImageJ [24].

To assess the effect of making leaf replicas on cellular morphology, micrographs were acquired from replicas of youngest fully developed leaves at Day 1 and 14 days later (Day 14) when they were fully developed adult leaves. Using the open-source image analysis program ImageJ (https://imagej.nih.gov/ij/), the major and minor axis of guard cells (length and width) were measured on fresh leaves under a brightfield microscope (Olympus IX-81, Olympus Corp, Tokyo, Japan) and on SEM-micrographs of tilted replicas. First, to assess growth, the means of the major (length) and minor (width) axis of each guard cell was measured in Day 14 replicas and were compared to those in Day 1 replicas. Next, the effect of making replicas on the leaf was tested by comparing the mean guard cell length and width in Day 14 replicas, between leaves treated with putty on Day 1 replicas and with no application. In addition, photographs of the abaxial surface of leaves and the whole plant were taken immediately before and after the replicas were made.

### Conventional sample preparation

Sample preparation was carried out according to a slightly modified protocol from Müller & Zechmann [8]. Small pieces (1mm^2^) of fully developed adult leaves (Day 14) taken around the midrib (box in Fig 1A) were fixed for 90 min with 2.5% glutaraldehyde in 0.06M Sorensen phosphate buffer at pH 7.2. After 4 washes in buffer for 10 min each, the samples were dehydrated for 20 min per concentration in a graded series of increasing concentrations of ethanol (50%, 70%, 90%, and 100%). After dehydration samples were critical point dried (Leica EM CPD 300; Leica Microsystems) with a customized program for tobacco leaves which took about 80 min (settings for CO_2_ inlet: speed = medium & delay = 120s; settings for exchange: speed = 5 & cycles = 18; settings for gas release: heat = medium & speed = medium). After critical point drying, samples were mounted on aluminum stubs with carbon tape and sputter coated with 10 nm iridium (Leica EM ACE 600, Leica Microsystems) and imaged as described above.

## Results

**Fig 2:**
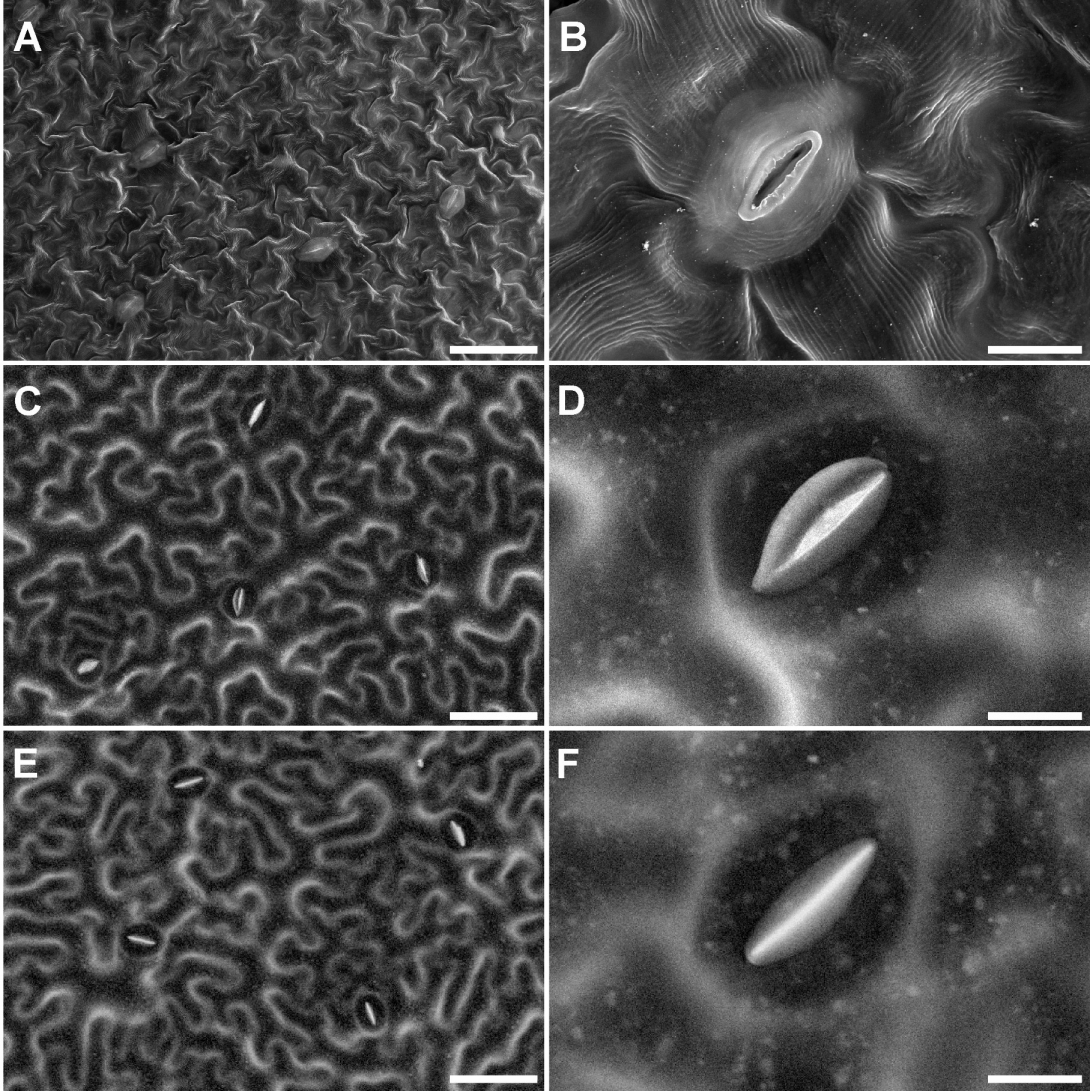
SEM micrographs showing surface structure of chemical fixed leaves and leaf replicas. (A) Overview of the surface of a leaf and (B) close up of a stomatal complex after chemical fixation, critical point drying, and sputter coating under high vacuum conditions. (C, E) Overview of the surface of a leaf and (D, F) close up of a stomatal complex from the replica. All images were taken from the center of the leaf as indicated by the black box in Fig 1. While images C and D feature youngest fully developed leaves (Day 1), images A, B, E and F feature adult leaves 14 days after the replica was taken (Day 14). Image C-F were taken from replicas taken from the exact same leaf at the beginning of the experiment (Day 1) and 14 days after a replica was taken from that leaf (Day 14). Images A and B were taken with the Versa 3D SEM at 30 kV while images C-F were taken with the TM-3030Plus at 15kV. Bars = 50 μm in A, C, and E, and 10 μm in B, D, and F.

Making leaf replicas did not cause any visible changes to the *Nicotiana benthamiana* leaves. Photographs of leaves taken before, immediately after, and 14 days after the replicas were made did not reveal any differences between leaves that were used to make replicas and those that were not (Fig 1A and 1B). Leaves treated using chemical fixation and critical point drying showed well preserved surface structures (Fig 2A and 2B). The outline of individual epidermis cells as well as guard cells, stomatal pore, and inner cell walls were clearly visible (Fig 2B). Shrinkage of epidermal cells was induced by chemical fixation and critical point drying and resulted in a wave-let like artifact on the cuticle, especially around guard cells and the middle of epidermal cells (Fig 2A and 2B). Wave-let artifacts were not found on the surface of replicas (Fig 2D and 2F).

**Fig 3:**
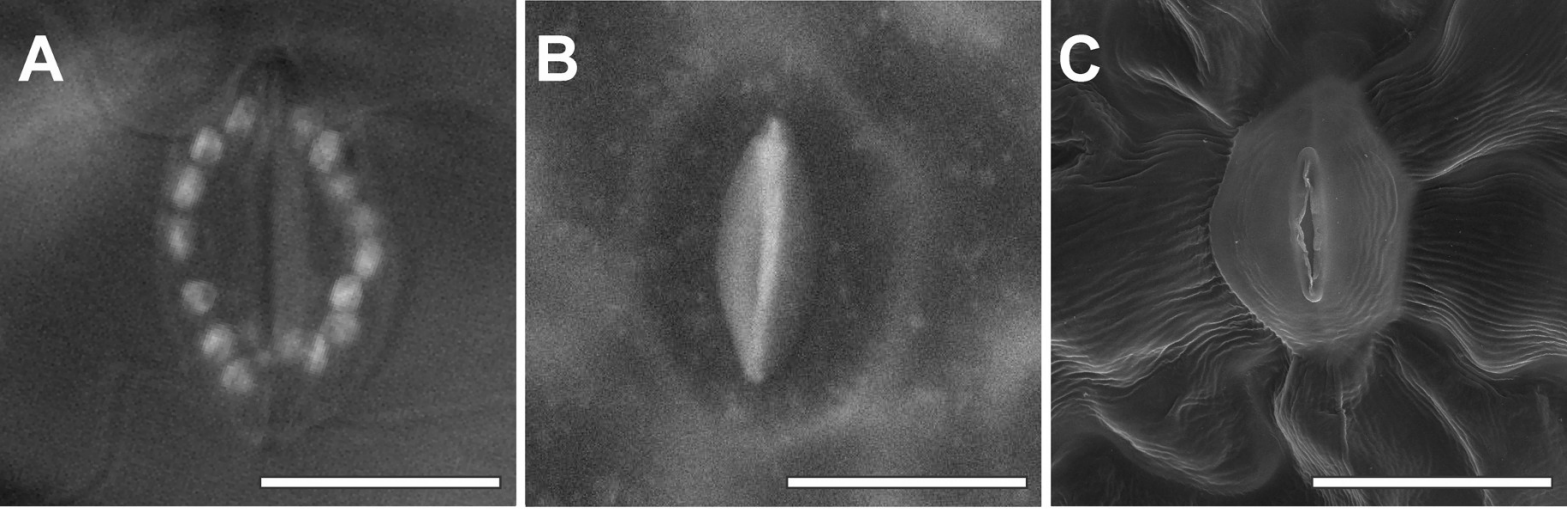
Comparison of differently prepared guard cells. Images show guard cells of fresh leaves under the light microscope (A), of a replica (B), and of a conventionally prepared sample (C) in the SEM. All samples derived from fully developed adult leaves (Day 14) close to the midrib as indicated by the black box in Fig 1A. Bars = 20 μm.

Replicas revealed similar surface information under SEM. The outline of epidermal cells as well as guard cells could be clearly identified on the replicas enabling measurement of guard cell size (Figs 2C–2F and 3). The outline of both guard cells was clearly distinguishable (Fig 2D). The size of guard cells did not differ significantly between fresh leaves under the light microscope and the replica under the SEM. Guard cells in conventionally fixed samples were 40% (length) and 38% (width) smaller than on fresh leaves (Table 1 and Fig 3), indicating shrinkage of the sample was induced by conventional sample preparation.

**Table 1:** Comparison of the size of differently prepared guard cells.

Guard Cells	Length (μm)	Width (μm)
**Fresh Leaf—Light Microscope**	32.1 ± 0.5^ns^	22.5 ± 0.3^ns^
**Replica—SEM**	32.3 ± 0.2^ns^	23.7 ± 0.2^ns^
**Conventional—SEM**	19.4 ± 0.3***	14 ± 0.3***

The size of guard cells of fresh leaves under the light microscope and on replicas under the SEM did not significantly differ. Notice the strong shrinkage of guard cells in conventionally prepared samples in the SEM when compared to fresh leaves and replicas. All samples derived from fully developed adult leaves (Day 14) close to the midrib as indicated by the black box in Fig 1A. Significant differences were calculated between the fresh leaves, replicas, and conventionally prepared samples with a t-test.

*** indicates significance at the 0.001 levels of confidence. ns = not significantly different. n = 45 for each data point.

Among Day 14 replicas no qualitative differences were found between leaves that were not used to make replicas on Day 1, and those that were (Table 2). However, though visually similar, significant differences in guard cell lengths were found of leaves with repeat impressions taken at day 1 and 14 (P<0.05) with average lengths changing from 29.2 ± 0.5 to 32.3 ± 0.2 μm between Days 1 and 14 (Table 2). No differences in guard cell width were found for these repeated measurements. When guard cell dimensions were compared among measurements acquired from the Day 14 leaf impressions for those with single versus repeated putty application, no significant differences were found (Table 2).

**Table 2:** Comparison of the size of guard cells on replicas between untreated and leaves treated with dental putty.

Guard cells		Length (μm)	Width (μm)
**A**	*Day 1*	29.2 ± 0.5^a^	23.0 ± 0.4^c^
	*Day 14*	32.3 ± 0.2^b^	23.7 ± 0.2^c^
**B**	*Day 14*	31.6 ± 0.3^b^	23.3 ± 0.2^c^

In experiment A the youngest fully developed leaves were treated with putty once (Day 1) and again 14 days later (Day 14) when they were fully grown adult leaves. In experiment B, comparable control leaves were only treated once with putty at the end of the experiment (Day 14). All samples were derived from an area close to the midrib as indicated by the black box in Fig 1A. Notice that there is no difference between the size of guard cells treated with or without putty at the beginning of the experiment, indicating that the application of the putty does not have long term negative effects on leaf surface morphology. Data are means ± standard errors. Significant differences were calculated using a one-way ANOVA in the statistical software JMP (SAS, Cary, NC). Different lower-case letters indicate significant differences at 0.01 level of confidence. n = 45 for each data point.

**Fig 4:**
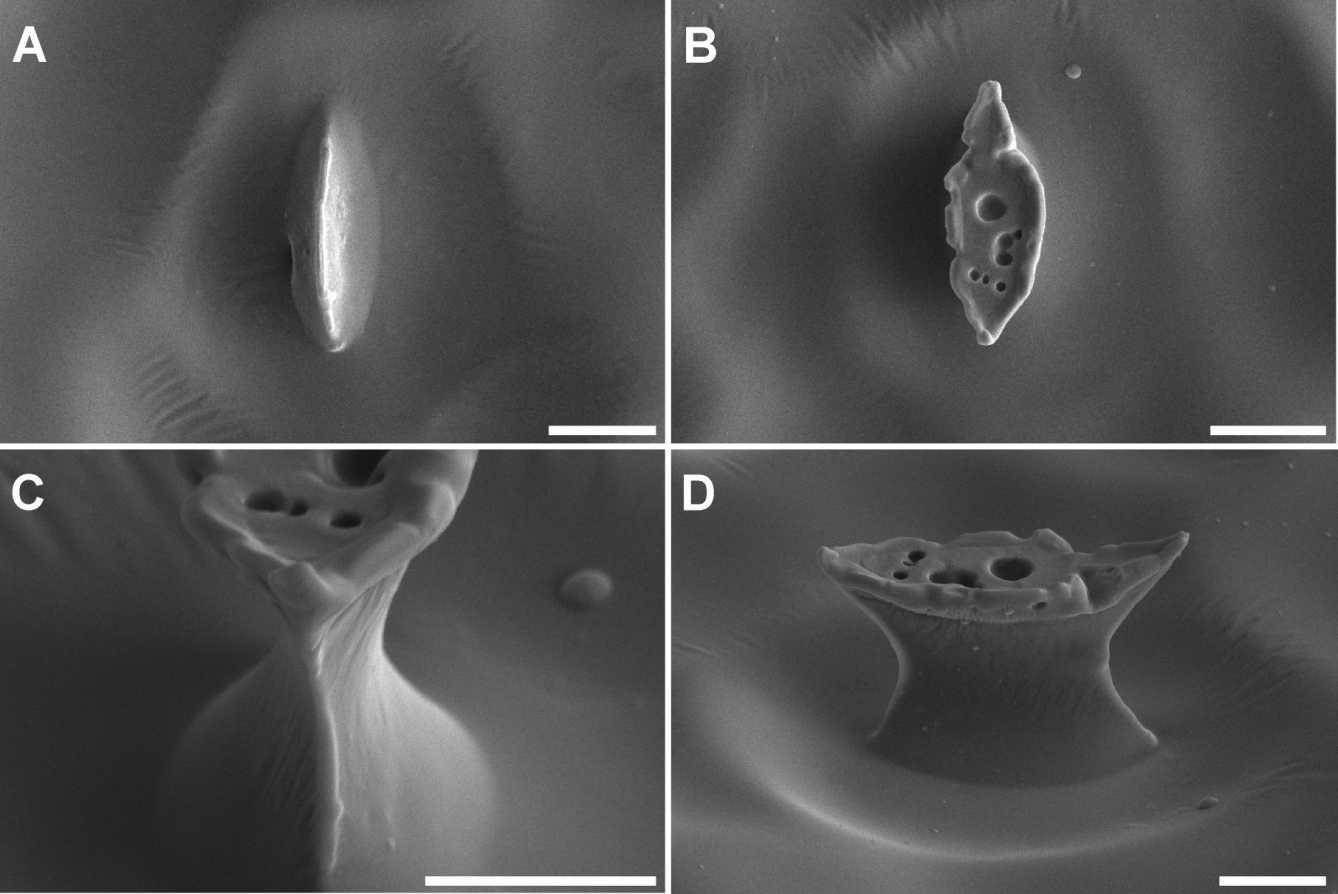
SEM micrographs of stomatal complex on leaf replicas. All images were taken from the center of the leaf, as indicated by the black box in Fig 1, from fully developed leaves (Day 14). Images were taken at 0 degree tilt angle showing a replica of (A) a closed stomata and (B) an open stomata and at 60 degree tilt angle showing protrusions on replicas of (C) the front and (D) the side of an open stomata. Images were taken with the Versa 3D SEM at 30 kV. Bars in A, B = 10 μm, C, D = 5 μm.

Replicas of open stomata showed intrusions into the sub-stomatal cavity, which did not occur on replicas of closed stomata (Fig 4). Tilting the replicas gave a clear view of the inter-guard cell space, allowing measurement of the stomatal pore at the thinnest part of that space, which is assumed to approximate *in vivo* stomatal aperture. Stomatal aperture width and length were measured where the protrusions’ dimensions showed a distinct mid-pore minimum (Fig 4C and 4D and Table 3). Parts of the sub-stomatal cavity may also be visible.

**Table 3:** Stomatal density and 3D dimensions.

SD	stomata mm^-2^	35 ± 2.1
**Pore length**	μm	10.4 ± 0.6
**Pore width**		2 ± 0.1
**Pore depth**		8.4 ± 0.6

Stomatal density (SD) from putty replicas and 3D aperture dimensions from micrograph measurements of dental putty intrusions into inter guard cell spaces. All samples derived from fully developed adult leaves (Day 14) close to the midrib as indicated by the black box in Fig 1A. Data are means ± standard errors. n = 7 replicas.

## Discussion

SEM is widely used to collect information about the surface, physiological state, and morphology of plant leaves [1–6, 8, 9]. To use SEM, samples have to go through several preparation steps to withstand high vacuum conditions and the energy of the beam. Preparation steps may include removal of leaves from the plant, fixation, dehydration, critical point drying and sputter coating [2, 8]. These steps can take several hours to a few days and lead to artifacts such as shrinkage of the cells.

Conventionally prepared leaves in this study showed evidence of cell shrinkage in the form of a wave-let structure of the cuticle, especially around guard cells and in the middle of epidermal cells. In addition to these qualitative artifacts, conventionally fixed guard cells in this study were 40% (length) and 38% (width) smaller than on fresh leaves. Replicas did not show signs of wave-let structure or shrinkage. Such artifacts may be avoided when leaves are observed at low vacuum conditions, ESEM, or at low temperatures after cryofixation, which do not require the above-described steps. In low vacuum or ESEM mode, however, artifacts are still very common when using such methods due to the removal of the leaf from the plant and high evaporation potential [2, 12, 14], which also limit SEM studies. Cryofixed cells are very sensitive to the beam which can cause cracking of the surface. This technique requires a high level of skill, expensive equipment [2, 3], removal of the leaves from the plant, and cannot be applied in the field. They are, therefore, prone to artifacts and invasive making them unsuitable for characterization of *in vivo* morphology or repeated investigations of sensitive structures.

The preparation of leaf replicas or leaf impression and investigation with SEM is a powerful alternative to conventional sample preparation [20–23]. Despite the advantages of requiring less skill and labor it is rarely used in SEM. The main reasons therefore are that the final results are affected by the use of organic solvents, material viscosity, environmental humidity, and mechanical damage [23] and that the application of some resins can even damage or kill the tissue [17, 21]. On top sample preparation can take up to one week and can require the removal of plant parts [20]. The method described in this study can be performed in less than 10 minutes, does not require any laboratory equipment, and does not damage the leaves. In comparison to all other leaf impression techniques described in the literature [20–23] the method used in this study is by far the fastest one and the only one that does not require any additional equipment for sample preparation. The obtained results by SEM investigations demonstrate that the production of replicas of leaf surfaces gave similar surface information when compared to samples prepared conventionally or by cryo-fixation [2, 3, 12], but without damaging the leaves or inducing apparent artifacts. SEM on the replicas allowed single epidermal cells and guard cells to be visualized after only a few minutes of sample preparation under low vacuum. Shrinkage of cells as described for conventionally fixed samples [2, 3, 5, 10, 11] was not observed on the replicas. The size of guard cells on replicas did not differ from fresh leaves demonstrating that the impressions capture the *in-vivo* state. The surface of the leaf replicas was comparable to the surface of leaves prepared by cryo-fixation and imaged at low temperature [2, 3, 12]. Additionally, the replicas showed high stability when exposed to the electron beam under high and low vacuum conditions. Cracking and hole formation as described for cryo-fixed leaves observed by SEM at low temperature conditions [2, 3, 12] were not observed on the replicas. Charging effects that have been described during low temperature SEM, low vacuum SEM, and ESEM [1–3, 5, 6, 12] did not occur when replicas were imaged under low vacuum conditions. Therefore, sputter coating replicas with a layer of conductive material as performed in other studies using leaf impressions [20–23] was not necessary in this study. For imaging samples at high vacuum conditions replicas were sputter coated to avoid image distortions caused by excessive charging. The addition of conductive materials such as silver or gold to the putty should eliminate the need for sputter coating for SEM-investigations under high vacuum and could be developed for future applications.

This method is highly favorable for plant physiological studies for several reasons. Non-invasive sampling without inducing artefacts, demonstrated here, allows for repeated sampling of the same leaf. For other methods, including other leaf impression techniques, leaves must be detached to be imaged by SEM [6, 8, 12, 20] making subsequent measurements of the same leaf impossible, and introducing nuisance inter-leaf variation to physiological studies. Replicas using this method allow for the creation of time-series with the same leaf, eliminating inter-leaf variations. Further, measurements from replicas are not affected by shrinkage artifacts, and capture the *in vivo* state. Replicas therefore lend themselves to plant physiological inference of, for example leaf gas exchange. Resolution of SEM was fine enough to detect small changes in guard cell size, thus providing a novel method for measuring cellular growth rates on living plants. Finally, replicas may be stored at regular room conditions and not lose their integrity over time, allowing for the creation of experimental archives.

Observation of the 3D structure of replicas makes valuable *in vivo* information about the complexity and diversity of stomatal structure more accessible [25]. Specifically, oblique angle viewing by SEM has allowed us to characterize the 3D structure of intrusions of the replica material through the stomatal aperture, which are created during replica formation. Past studies have gained valuable insight into difficult-to-access plant development and surface physiology using replica-based techniques [15–23, 25–27]. However, previous studies were practically constrained by SEM technology to a perspective of replicas’ external surface [18].When viewed top-down, as in several previous studies [17, 19–23, 26, 27], replica intrusions may have obscured the stomatal pore, which is by definition the narrowest opening through which gas must diffuse [18]. Oblique angle viewing here revealed that the top surface of putty intrusions is sometimes considerably larger than the stomatal pore in open, but not closed stomata, potentially affecting the results of physiological inferences [25–27]. Some studies have used secondary reproductions (‘double-negatives’) to reproduce the external appearance of stomata [20–23], possibly compounding any errors produced in reproduction. We argue that oblique angle viewing of a primary reproduction of the aperture by SEM is advantageous as it reveals the 3D structure of the stomatal interior, rather than the exterior, without compounding reproduction error. This allows for location of the minimum opening, which may vary between species, and measurement thereof. Stomatal pore dimensions reported here are the most direct characterization of the minimum aperture reported using replica techniques, and may be used to characterize diffusive limitations on leaf gas exchange. Additionally, and unique among impression-based techniques, oblique angle viewing of replicas may also allow estimates of aperture depth, a stomatal feature normally obtained from cross-section of leaves coupled with transmission electron microscopy [29]. Further, replicas may serve as a basis for 3D reconstruction of complex and diverse pore morphology and more advanced models of gas diffusion through stomata. Such data together with data on SD can then be used to improve calculation of important functional features of leaves such as stomatal conductance and water use efficiency [30, 31] especially from repeated measurements of living specimens with limited effect. Collecting data of stomatal morphology and density from replicas and SEM will open up avenues for further plant physiological investigations that requires non-destructive sampling and or repeated *in vivo* measurements.

## Conclusions

Stomata are complex and diverse 3D plant organs of great ecological and economical importance. Replicas are well suited to investigate the surface of leaves by SEM and calculate SD, but also to sub-surface 3D morphology like pore width. The method described in this study is highly accessible in that it is inexpensive, simple to apply, can be performed in the field, and results can be obtained in a few minutes. For those who do not have access to the required SEM technology or expertise, stable replicas can be sent to collaborators. The chemicals involved are non-toxic and it requires fewer expensive instruments than other SEM based and leaf impression techniques (e.g. high-pressure freezer, critical point dryer, fume hood, ovens, sputter coater, etc.). Additionally, this method is non-invasive and can be repeatedly performed on the same leaf without damaging the surface or affecting cellular morphology, potentially on rare or delicate samples such as desiccated herbarium specimens. The ultrastructural properties of the replicas are comparable to conventionally prepared samples and even superior in some respects, as shrinking of the cells is not an issue. Tilting of the replicas enabled an unobstructed view and improved measurements of the stomatal aperture dimensions in 3D. As data on *in vivo* stomatal pore morphology such was width, area and depth are difficult to obtain, the replicas may be valuable method to evaluate these structural attributes as an inexpensive alternative to other microscopy methods that require tissue harvesting and extensive preparation.
